# Cost Analysis of Outpatient Colectomy in a Tertiary Center: A Projected Medico-Economic Evaluation

**DOI:** 10.1177/11786329241284400

**Published:** 2024-09-24

**Authors:** Fabio Agri, William Möller, Philip Deslarzes, Charles André Vogel, Dieter Hahnloser, Martin Hubner, Nicolas Demartines, Fabian Grass

**Affiliations:** 1 Department of Visceral Surgery, Lausanne University Hospital CHUV, University of Lausanne (UNIL), Lausanne, Switzerland; 2 Department of Administration and Finance, Lausanne University Hospital, Lausanne, Switzerland; 3 General Direction, Lausanne University Hospital, Lausanne, Switzerland

**Keywords:** Prospective payment system, diagnosis related groups, outpatient colectomy, cost-revenue, financial incentives, 30-day readmission, post-operative complications

## Abstract

**Aim of the study:**

Short stay processes are incentives to unburden chronically stressed healthcare systems. The aim of this study is to analyze financial implications of day admission (DAS) and outpatient strategies for colon resections in a prospective payment system (PPS) using Diagnosis Related Group (DRG) coding.

**Methods:**

Consecutive patients undergoing left and right colonic resections between January 1, 2019 and December 31, 2020 were included. Medico-economic evaluations of the virtual outpatient and day admission surgery groups based on predefined criteria were compared to the identical group of patients who underwent surgery in the actual traditional inpatient setting. In a second step, postoperative complications of the virtual outpatient group were assessed. Cost-revenue analysis was performed using a micro-costing approach including direct medical costs.

**Results:**

Overall (N = 257), 97 (37.7%) colectomies would have been potentially eligible for an outpatient strategy. The global costs of the actual inpatient strategy totaled USD 3 634 392 with a global revenue of USD 3 571 069, corresponding to a cost coverage rate of 98%. The result of the virtual DAS strategy would have been a net loss of USD 15 800 (coverage rate of 99%) due to 4 low length of stay outliers triggering a reimbursement reduction and preventing a positive net result of USD 16 208. The pilot reference outpatient case’s revenue and cost amounted to respectively USD 7479 and USD 6911 (cost coverage of 108%).

**Conclusion:**

From both any given hospital and healthcare system point of view, elective outpatient colectomy for selected patients is the most cost-saving option. However, in a prospective payment system implemented to avoid bad incentives, the latter can unintentionally disadvantage best performing hospitals and impede widespread adoption of high-value strategies.

## Introduction

As a consequence of a continuous increase in healthcare costs over the past decades, different hedging strategies were developed to reverse the trend. Switzerland (CH) ranks among the top countries in the world in terms of healthcare expenses in relation to gross domestic product GDP.^1^ This situation calls for counterbalancing strategies, and short-stay processes are increasingly recognized as a model to follow. While enhanced recovery pathways (ERP) were widely adopted a decade ago with a beneficial impact on postoperative complications and, as a consequence, reduced length of stay (LOS).^2,3^ Day admission surgery (DAS) strategies (patient admission the morning of surgery) were implemented to reduce unnecessary costs before surgery. Moreover, according to several recent studies, short stay processes decrease nosocomial infections and thromboembolic risks.^3,4^ Under the incentive to optimize further, the concept of ambulatory care has been promoted in Switzerland in more recent years. As per January 1st, 2023, a list of 18 procedure groups applies throughout the country, and these operations are reimbursed only when performed in an outpatient setting, unless special conditions require inpatient treatment.^5^ Colectomies are not yet included in the list, despite demonstration of feasibility of ambulatory colectomy in highly selective and dedicated settings.^6-9^ This strategy may apply to up to 30% of selected patients according to a French pioneer group.^10^ Recently, our group has developed a simple and comprehensive score to help select patients to undergo safe outpatient colectomy with a risk of hospital readmission of less than 20%.^11^ While the clinical score requires prospective validation, detailed analysis of financial implications in a prospective payment system (PPS) is important.

Introduced in Switzerland in 2012, the national PPS based on diagnosis related groups (DRGs) for inpatients sets incentives to treat patients cost-efficiently. This case-based reimbursement system implies a grouping algorithm, using diagnostic and procedure codes, to allocate cases to their respective DRG. In the outpatient sector, services are billed based on a national fee-for-service pricing structure.

The purpose of the current study is to analyze financial implications and incentives associated with different patient management strategies (in- and out-patient) from both the hospital and healthcare system point of view.

## Methods

Monocentric retrospective cohort study including consecutive patients who underwent elective left and right colonic resections at the Lausanne University Hospital CHUV between January 1st, 2019 and December 31, 2020. No patient was excluded from the analysis unless general consent was not obtained. Diagnosis Related Groups (DRGs) were obtained for each case combining diagnostic codes of the 10th edition of the German modified international classification of disease (CIM-10-GM), procedures codes (CHOP codes based on the Swiss classification of interventions) as well as demographic holdout data such as age, gender and type of admission. Each DRG has its resource utilization score called cost weight (CW). The fixed amount per case to be billed is thus obtained by multiplying the CW by the hospital’s base rate (BR). The latter represents a fixed conversion factor in Swiss francs (CHF) that is usually renegotiated each year.^12^

**Figure 1.**
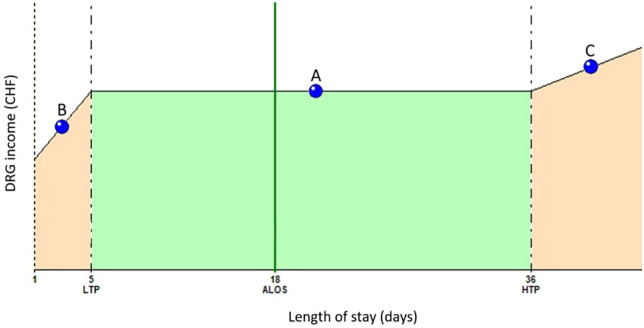
DRG chart: Inlier and outlier reimbursement. A case falling in the above DRG chart is an inlier if its length of stay (LOS) falls in between the lower (low trim point, LTP) and upper (high trim point, HTP) limit set for that DRG: (A) example of an inlier: There is no deduction or surcharge on the base amount of the invoice, (B) example of a low outlier: There is a deduction taken from the inlier fee of the considered DRG, and (C) example of a high outlier: There is a surcharge to be added to the inlier invoice (fee) sent to the payer. Abbreviations: ALOS, average LOS; DRG, diagnosis related group; HTP, high trim point; LTP, low trim point.

The national PPS ensures fixed fees for length of stays (LOS) statistically defined as “normal” for the DRG considered (inliers). Outlier LOS for the same DRG trigger a reimbursement amount adjustment. Outliers are patients displaying atypical characteristics relative to other patients in the same DRG. While *high LOS outliers* trigger a per diem increase in the base reimbursement amount for the hospital, *low LOS outliers* trigger a financial deduction taken from the base reimbursement amount provided for inliers in that DRG (Figure 1).

In the outpatient sector, services are billed based on a national fee-for-service pricing structure. In this case, outpatient medical and nursing services, specific analyses (ie, labs), medications and required medical equipment are billed by the provider. Each service is assigned a certain number of points in the pricing structure and the monetary value of the point is renegotiated regularly.^13^ Laboratory analysis, medications and medical equipment are billed on the basis of an official tariff established by the Swiss Federal Office of Public Health (FOPH). Medical equipment not disposing of an official tariff by the FOPH can be billed at cost price.^14^

Financial amounts were obtained in Swiss francs (CHF) and then converted to U.S. dollars (USD). The exchange rate used was USD 1 = 0.88 CHF, the official rate on March 5, 2024.

All patients received peri-operative care according to enhanced recovery after surgery (ERAS) principles, which were fully implemented in the institution 12 years ago.^15^ While ERAS focuses on pre-, intra-, and postoperative items to improve recovery and, consequently, decrease postoperative LOS, logistical investment and execution of DAS were only recently deployed in our institution.^16^ Therefore, during the study period, patients scheduled for elective surgery were still admitted the day prior to the operation.

Three settings were analyzed: the actual inpatient setting as represented by the study cohort (inpatient group), a virtual DAS setting assuming that all study patients were admitted to the hospital at the day of surgery (DAS group), and a virtual outpatient setting considering only selected eligible patients according to predefined criteria.^11^ These criteria include 7 factors: sex; minimally invasive surgery; American society of anesthesiology (ASA) score; wound class; ileostomy; surgical duration; perioperative intravenous fluid. Each item receives a risk score, and according to a total score below a critical threshold (⩽2 points), the risk of experiencing an adverse outcome is estimated at <20% (post-operative ileus, intra-abdominal abscess, anastomotic leak, and readmission).^11^ For descriptive purposes of the cohort, further patient characteristics including age, immunosuppressive therapy and age-adjusted Charlson comorbidity index (a-CCI) were also collected.^17^

Economic evaluations of the virtual outpatient and DAS groups were compared to the identical actual inpatient group (study cohort).

In a second step, postoperative complications and 30-days readmission rate of patients in the outpatient group were assessed to evaluate whether an outpatient colectomy would have been feasible without readmission based on postoperative complications. Each postoperative complication was classified using the Clavien-Dindo et al classification of surgical complications.^18,19^

The single ambulatory pilot case of the cohort was operated by institutional surgeons, outsourced to an affiliated lean structure, the ambulatory surgery center (ASC).

Cost analysis was performed using a micro-costing approach,^20^ including direct medical costs and revenue (fee). The costs and revenue were extrapolated for the DAS group. To extrapolate the costs of the DAS strategy, an evaluation of the average cost per pre-operative day spared was carried out. For this purpose, medical (surgeons and anesthetists), nursing and hotel (room and food) costs for a sample of 100 cases with a surgical DRG G18B (*intervention on intestine and colon or other intervention on the stomach, œsophagus and the duodenum, without prior radiotherapy*) were used to estimate the average cost related to the preoperative admission day as this is the most common DRG for colorectal patients undergoing a surgical procedure.

Billable procedures for the outpatient group were obtained through the real use case. Mean costs, revenue, and margin generated for the hospital were compared between the actual inpatient group, the virtual DAS group with and without low LOS outliers, and the virtual outpatient group. Student’s *t*-test, two-sided 95% CI’s, was used for the 3 pairwise comparisons of the different strategies respective mean costs and margins: Actual inpatient versus DAS with low LOS outliers, DAS with low LOS outliers versus DAS without low LOS outliers, and DAS without low LOS outliers versus actual inpatient. The cost-revenue analysis in the outpatient group was extrapolated from a single outpatient laparoscopic right colectomy pilot case. Therefore, to compare the costs and margin single data of the outpatient strategy with the mean costs and margins of each of the other strategies, a one-sample *t*-test was conducted.^21^ Excel Office 2016 descriptive statistics tools and Statistical Package of the Social Sciences (SPSS) versions 29.0 were used. A *P*-value <.05 was considered significant.

The study protocol was approved by the local institutional review board (*Commission* cantonale d’éthique de la recherche sur l’être humain CER-VD 2023-00940). Written informed general consent for research was obtained from all study participants.

## Results

**Figure 2.**
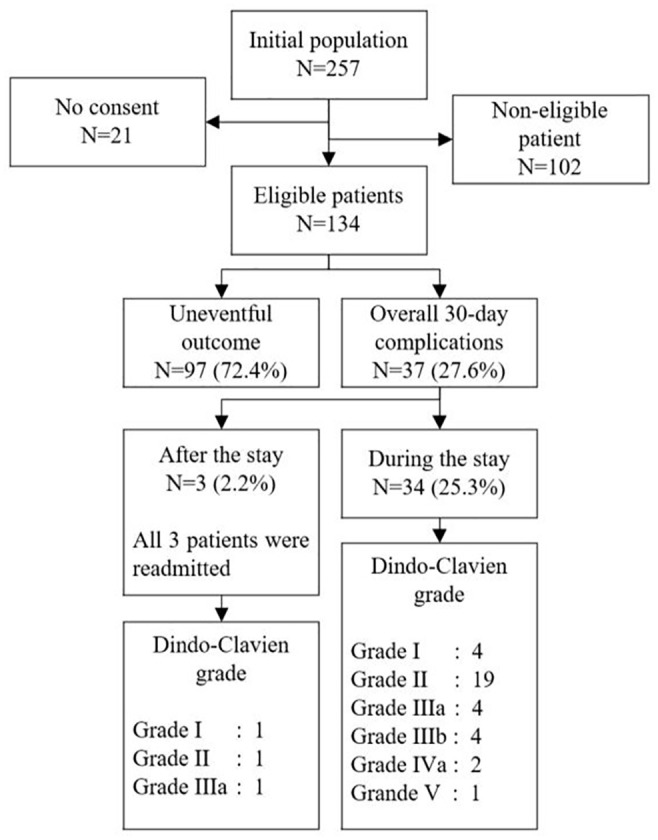
Study flow chart.

Out of 257 elective colectomy procedures over the 2 years period, 21 patients did not consent to reuse their data or engage in clinical research. According to the institutional selection criteria, 134/257 (52.1%) patients would have been eligible for an outpatient strategy, representing the virtual outpatient group. The virtual outpatient group consisted of 51% female patients with a mean (SD) age of 61 (15) years, a mean ASA score of 2.1 (0.5), with 14% of patients being on immunosuppressive therapy at the time of surgery, and a mean a-CCI score of 2.7 (2.2) points. Of these 134 patients, 97 (72.4%) had an uneventful postoperative course and were hence considered as potential outpatients based on postoperative complication profiles alone (Figure 2). The latter group had 48% of female patients, a mean (SD) age of 59 (16) years, a mean ASA score of 2.2 (0.6), 13.4% (13/97) were on immunosuppressive therapy, and the a-CCI score was 2.6 (2.1) points. Comparing the group of patients without complications (n = 97) versus the group of patients with complications (n = 37): male patients: 48/97 (49%) versus 23/37 (62%), *P* = .246, immunosuppression: 13/97 (13%) versus 7/37 (19%), *P* = .426, age: 60 ± 16 versus 62 ± 16, *P* = .18, ASA 2.2 ± 0.6 versus 2.1 ± 0.5, *P* = .34, a-CCI score 2.6 ± 2.1 versus 3.4 ± 2.7, *P* = .04.

The proportion of right colectomy (including ileocecal resection (n = 10), right colectomy (n = 43) and extended right colectomy (n = 4)) with complication was 19/57, and the proportion of left colectomy (including left colectomy (n = 6), extended left colectomy (n = 1), sigmoïdectomy with anastomosis (n = 53), and sigmoïdectomy-Hartmann (n = 6)) with complication was 10/66 (*P* = .02).

Of the entire cohort, 97/257 (37.7%) would hence have been retrospectively eligible for an outpatient strategy.

The most frequent indication for ambulatory colectomy was colon cancer (66.4%), and the most frequently performed procedure was left colectomy (54.5%) (Table 1). The single outpatient case had a right colectomy for diverticular disease.

**Table 1.** Demographics and surgical characteristics.

Eligible population	N = 134 (%)
Demographics	
Age (years)^a^	61 (15)
Sex ratio (M:F)	62:72
Indication	
Cancer	89 (66.4)
Sigmoïd diverticulitis	30 (22.4)
Inflammatory bowel disease	11 (8.2)
	3 (2.2)
Sigmoïd volvulus	1 (0.7)
Procedure	
Laparoscopic left colectomy	73 (54.5)
Laparoscopic right colectomy	58 (43.3)
Laparoscopic total colectomy	3 (2.2)

a Mean (SD).

The most represented DRG is the G18B (*procedures on the small intestine and colon or other procedures on the stomach, esophagus and duodenum without radiation therapy*) with an inlier CW of 1.793 point and a CW reduction of 0.605 point if the patient hospital stay was less than 2 nights (low LOS outlier) in 2019 and a CW of 1.78 point and a CW reduction of 1.123 point if the patient stay was less than 2 nights in 2020. The amount of the fixed conversion factor in CHF, corresponding to the hospital’s base rate (BR) stayed unchanged at CHF 10 650 (USD 12 035) for both years.

**Figure 3.**
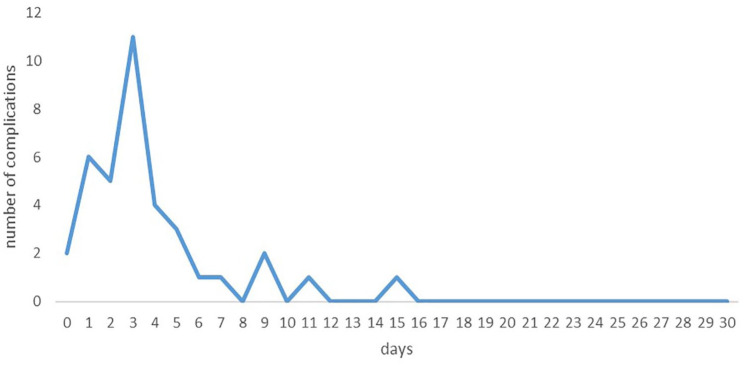
Time of diagnosis of postoperative complications.

The overall complication rate of the virtual outpatient group (n = 134) at 30 days was 27.6%. Of the 37 patients presenting with at least 1 complication, 34 (91.9%) were diagnosed during their hospital stay and 4 (10.8%) while they were already discharged, and all of them were readmitted. Two third of the complications appeared after POD 2 with a peak at POD 3 (Figure 3). The ambulatory pilot patient did not present any complications.

Among patients presenting an adverse event (N = 37), 28 (75.7%) were managed conservatively and 9 (24.3%) patients underwent a reintervention (Table 2). Of these latter, 3 underwent laparotomy (2 for small bowel obstruction and 1 for anastomotic leakage) and 1 underwent laparoscopy (anastomotic leakage), 2 patients were treated by colonoscopy (gastrointestinal bleeding), 2 patients underwent interventional radiology (1 pleural effusion and 1 deep surgical site infection) and finally 1 patient underwent surgical incisional abscess drainage.

**Table 2.** Complications and management strategies.

Complication	N = 134 (%)	Management		
During the stay		Conservative (N)	Interventional (N)	
Small bowel obstruction	15 (11.2)	13	2	
Surgical site infection	8 (6)			
Superficial incisional	2 (1.5)	2	-	
Deep incisional	1 (0.7)	-	1	
Organ space^a^	5 (3.7)	3	2	
Abdominal wall (incisional) hematoma	3 (2.2)	3	-	
Lower gastrointestinal bleeding	2 (1.5)	-	2	
Urinary retention	2 (1.5)	2	-	
Cardiac rythm disorder	1 (0.7)	1	-	
Pleural effusion	1 (0.7)	-	1	
Urinary tract infection	1 (0.7)	1	-	
Pneumonia	1 (0.7)	1	-	
At home				Readmitted
Surgical site infection	2 (1.5)			
Superficial incisional	1 (0.7)	1	-	Yes
Deep incisional	1 (0.7)	-	1	Yes
Small bowel obstruction	1 (0.7)	1	-	Yes
**Total**	**37 (27.6)**	**28 (20.9)**	**9 (6.7)**	**3 (2.2)**

a Including 2 cases of anastomotic leakage.

### Cost analysis

#### Inpatient strategy

The national (CH) mean (SD) length of stay (LOS) for the DRG codes of the entire cohort was 9.6 (4.63) days, whereas the mean (SD) LOS of the 134 virtual outpatients was 7.8 (5.8) days.

Overall costs for the entire cohort amounted at USD 3 634 392, while the global revenue totaled USD 3571 069. Hence, for the entire cohort, the hospital was confronted with a net loss of USD 63 323, corresponding to a cost coverage rate of 98%.

### Virtual DAS strategy

The costs related to the day before the intervention and thus saved within the virtual DAS strategy was valued at USD 611.8. Overall, global costs of the virtual DAS exercise amounted at USD 3 552 414 and global revenue at USD 3 536 614. The global result is thus a virtual net loss of USD 15 800, corresponding to a cost overage rate of 99%. Although the virtual DAS group generated 4 low outliers responsible for a total loss of revenue of USD 34 455, the overall savings of USD 81 979 (134 × 611.8) limited the loss. Assuming the 4 low outliers were avoided by keeping them intentionally 1 day longer in the hospital, the loss of revenue (USD 34 455) could have been avoided at a price of 4 extra-days at USD 611.8 (USD 2447). The overall projection would have been a positive net result of USD 16 208. In this latter case, the coverage would have reached 101%.

### Outpatient strategy

Cared for in a dedicated ambulatory surgery center (ASC) affiliated to the main institution, the revenue of the single reference case was USD 7479, and the costs amounted to USD 6911, corresponding to a cost coverage of 108%. If the same case was entirely handled in the main institution using the same resources also available for inpatient procedures, the same revenue would have been generated, but costs as high as USD 8630, resulting in a net loss of USD 1151 and thus corresponding to a cost coverage of 87%.

Only considering postoperative complications, 97 of the initially screened cohort (257 patients, 37%) could have benefited from an outpatient strategy. Extending the ambulatory strategy to 37% of the population eligible for an outpatient strategy and proposing DAS to the other 63% allows a coverage rate of 101% if low outliers are considered among the DAS group and 102% without reduced fees for low outliers. Table 3 details the net results and cost coverage rate of the different strategies.

**Table 3.** Net results and cost coverage of different strategies for colectomies.

(A) Per case financial result							
				Inpatient “actual” strategy Mean (SD)	Inpatient « hypothetical » DAS strategy Mean (SD)		Outpatient « actual » strategy (ASC)
					With low outliers	Without low outliers^a^	1 Case index
Costs (USD)				27 122 (15 337)	26 511 (15 337)	26 528 (15 340)	6911
Revenue (USD)				26 650 (13 360)	26 392 (13 239)	26 709 (13 521)	7479
Net result (USD)				–472 (8648)	–118 (8440)	198 (8709)	568
*Coverage rate (%)*				*98.3*	*99.6*	*100.7*	*108.2*
(B) Overall financial result							
N = 134	100% Cases inpatients actual strategy	100% Cases “hypothetical” DAS strategy		Outpatient combined with “hypothetical” DAS strategy			
		With low outliers	Without low outliers^a^	37% Of outpatient cases (ASC)		63% Of DAS cases	
Costs (USD)	3 634 392	3 552 414	3 554 860	345 550		2 226 924	
Revenue (USD)	3 571 069	3 536 614	3 571 068	373 950		2 216 928	
Net result (USD)	–63 323	–15 800	16 208	28 400		9996^b^	
				18 404**			
*Coverage rate (%)*	*98.3*	*99.6*	*100.5*	*100.7* ^c^			

Abbreviation: ASC, ambulatory surgical center affiliated to the main university hospital center (CHUV).

a Intentionally keeping the 4 patients, which generated low outliers (reduced fee), 1 extra day.

b The mean revenue including reduced fees due to low outliers was used for the calculation of the net result (USD −9996). The net result would have been positive (USD 15 204) if the mean revenue of the “hypothetical” DAS strategy without low outliers was used.

c The coverage rate would have been of 101.7% if the hypothetical DAS strategy without low outliers was used combined to outpatient strategy, or a net result of USD 43 604 (USD 28 400 + USD 15 204) instead of USD 18 404** (USD 28 400 − USD 9996).

### Comparison of strategies

The mean costs of the actual inpatient strategy were USD 541 (95% CI: −2720 to 3805, *P* = .7443) compared to those of the DAS strategy with low LOS outliers. The mean costs of the DAS strategy with low LOS outliers were USD −16 (95% CI: −3280 to 3250, *P* = .9923) compared to those of the DAS strategy without low LOS outliers. The mean costs of the DAS strategy without low LOS outliers were USD −525 (95% CI: −3790 to 2740, *P* = .7517) compared to the actual inpatient strategy. Compared to the outpatient strategy, the mean costs of each of the three strategies (actual inpatient, DAS with low LOS outliers and DAS without low LOS outliers) were significantly higher (*P* < .0001).

The mean margin of the actual inpatient strategy was USD −310 (95% CI: −2130 to 1500, *P* = .7343) lower than that of the DAS strategy with low LOS outliers. The mean margin of the DAS strategy with low LOS outliers was USD −280 (95% CI: −2105 to 1545, *P* = .7631) lower than those of the DAS strategy without low LOS outliers. The mean margin of the DAS strategy without low LOS outliers was USD 593 (95% CI: −1250 to 2440, *P* = .5275) higher than the actual inpatient strategy. There was no significant difference between the mean margin of each of the 3 strategies (actual inpatient, DAS with low LOS outliers, and DAS without low LOS outliers) and the outpatient strategy.

## Discussion

According to this retrospective analysis of clinical outcomes, 38% of all comers undergoing elective colectomy within a standardized enhanced recovery after surgery (ERAS) pathway could potentially be eligible for an outpatient setting.^11^ From a medico-economic standpoint, an outpatient strategy outsourced to a dedicated lean ASC structure represented the best option from both the hospital and the healthcare perspective. While waiting for prospective validation of the score, this study reveals a need to eliminate bad incentives, which prevent innovation toward cost-saving ambulatory colorectal surgery.

Short stay processes are ways to unburden chronically stressed healthcare systems. Enhanced recovery pathways like ERAS, day admission surgery (DAS), outpatient strategies and the use of connected tracking solutions for post-operative remote follow up are ways to reduce in hospital stay.^22^ While ERAS and tracking devices focus on postoperative follow-up and recovery, DAS was implemented to avoid unnecessary pre-operative overnight stays.^23^ Taken together, selected patients may be eligible for an ambulatory strategy.

Feasibility of outpatient colectomy has already been demonstrated in groups of highly selected patients.^9,10^ Our group developed selection criteria among a large bi-institutional cohort to help select patients in the immediate postoperative period for safe discharge.^11^ However, a projected economic evaluation within a prospective payment system has not yet been performed.

In the present series, most complications were diagnosed after 48 hours, but within 5 postoperative days, which is consistent with previous publications.^24,25^ This strongly suggests the deployment of a strategy for early detection of complications occurring within the first days after discharge. In our study, right colectomy was associated with more complications compared to left colectomy. This finding corroborates our previous institutional experience, confirming that patients undergoing right colectomy are more prone to complications, especially postoperative ileus.^26^ This must be considered when offering an outpatient strategy to these patients. A connected tracking solution was available for inpatients and outpatients after discharge, allowing the medical team to secure patient surveillance without multiplying medical visit and enabling rapid and coordinated action in the event of a complication.^22^ The institutional connected tracking mHealth app allows for active search of specific post-surgical complications through a questionnaire available on patient’s smartphone the day following hospital discharge for a duration of 7 days after colorectal surgery. All items are closed questions with predefined answers and recommendations. In case of an adverse event, alerts are automatically generated by the patient’s response. In parallel, a dedicated center of telemedicine was purposefully created for 24/7 availability and following a predefined alert algorithms.^22^

Daily phone calls, nursing or medical visits at patient’s homes or in hospital represent other solutions to consider. However, mobile app follow-up solutions present the advantage of delivering a convenient, patient-centered care solution with a high degree of satisfaction and cost-effectiveness.^22,27,28^

Avoiding unnecessary hospital exposure decreases the risk of nosocomial adverse events, contributes to patient satisfaction and may be profitable provided there is adequate follow-up.^3,4,29-34^ Successful short stay processes require proper patient selection, standardized perioperative care, and oiled logistics including remote monitoring solutions.^10,35,36^ Achieving an adequate level of competence in these domains requires a substantial investment, which is gradual for an institution. Given the importance of elements at stake for all stakeholders, the payment system must promote this transition by providing right incentives or at least not hindering its implementation by counterproductive incentives. The present study however shows that best performing hospitals deal with inadequate financial mechanisms. Despite widespread implementation of short stay processes (day admission surgery combined with telemedicine solutions), the generation of low outliers (LO) penalizes successful hospitals, thus preventing evolution of practices toward an ambulatory strategy. For example, in the present series, implementation of the DAS strategy would have generated a positive margin only by intentionally keeping the 4 LOs 1 extra day. The choices here were to either keep the 4 patients 1 extra day each to reach an overall positive margin of USD 16 208 or end up with a negative overall margin of USD 15 800 with early discharge. Of note, later days are less cost-intensive than early days of a hospital stay.^37^ For this reason, the cost of the extra day was assumed identical to the cost of the day before the intervention.

The projected scenario of 37% outpatient procedures combined with 63% DAS strategies reduces the overall costs by one-third compared to the DAS strategy alone for the 134 patients, corresponding to a projected sparing potential of nearly CHF 1 million for the healthcare system. Ambulatory surgery may represent a promising strategy for hospitals and in particular for academic medical centers to seek operational efficiency and financial gains. The significant economic impact of an outpatient strategy reported in the present study was based on a pilot case outsourced to an affiliated ambulatory surgical center (ASC). ASCs can operate at lower costs compared to main hospital facilities.^38-41^ If the outpatient pilot case was carried out in the main hospital, a deficit of USD 1151 instead of a gain of USD 568 would have been the net result. The institutional ASC focuses on outpatient services and therefore produces lower costs compared to the large, complex, and around the clock academic medical center.

It is important to analyze and correct without delay financial incentives to motivate best performing hospitals to invest in innovative strategies. Breakthrough innovations such as ERP or connected tracking may be corrective elements for bad incentives. Low outlier cases produced by an improved workflow process should be rewarded by inlier fees instead of generating financial losses. Consequently, the fee reduction would be lowered or even eliminated and ban incentives to keep the patient another unnecessary day, ultimately promoting patient turnover, enhancing hospital profit and costs-savings in the health care system. Furthermore, through the mechanism of annual fee adjustment based on previous years’ costs, best performers could inspire more efficiency on a national scale.

## Limitations

The study included consecutive patients undergoing elective colectomy over the study period without predefined sample estimates. Given the retrospective nature of the study, information bias is inherent. An overestimation of a successful outpatient strategy cannot be excluded. Indeed, we virtually applied an outpatient strategy to a preselected subset of patients with favorable baseline characteristics who underwent uneventful minimally invasive surgery. In reality, the outpatient population may obviously also present complications. In this case, their management would generate an additional outpatient or inpatient bill. This is advantageous from the hospital’s point of view, as complications in this context are reimbursed. On the other hand, complications following an inpatient stay also generate outpatient bills. However, they typically lead to a merged unique DRG for the 2 stays, thus not necessarily generating reimbursement related to a second bill. In this case, the financial risk is transferred from payers to providers.

Furthermore, the cost-revenue analysis has several limitations. First, it focused on direct medical costs and did not consider potential outpatient costs for inpatient strategies other than those directly related to the day of surgery for the outpatient strategy. However, the number of scheduled outpatient visits post-discharge does not differ between inpatient and outpatient strategies, with a scheduled control visit 2 to 4 weeks post discharge. This is possible thanks to the institutional connected tracking solution, helping to avoid additional visits for outpatients compared to inpatients.^22^ Second, cost-revenue analysis in outpatient group was extrapolated from a single outpatient laparoscopic right colectomy pilot case, which would have been grouped in the DRG G18B if it had been carried out in stationary mode. This single estimate was considered as being measured without error. The “simple” colectomy cases tend to fall into DRG G18B, which also represented the most frequent DRG of the actual strategy. This puts into perspective the uncertainty not considered in this single estimate. Also, due to the exploratory nature of our study, the comparisons were based on a two-sided 95% CI. Third, the study is based on hypothetical scenarios, which need confirmation in the real clinical setting. Finally, opportunity costs were not considered.^42^

## Conclusions

This study provides further evidence that colonic resections may be suited for outpatient management provided strict patient selection. If deployed in a dedicated lean structure mastering the concept of ERP, this strategy may be carried out at lower costs. However, in order to successfully and sustainably implement short stay processes, financial incentives must be encouraged.

## Supplemental Material

sj-docx-1-his-10.1177_11786329241284400 – Supplemental material for Cost Analysis of Outpatient Colectomy in a Tertiary Center: A Projected Medico-Economic Evaluation Supplemental material, sj-docx-1-his-10.1177_11786329241284400 for Cost Analysis of Outpatient Colectomy in a Tertiary Center: A Projected Medico-Economic Evaluation by Fabio Agri, William Möller, Philip Deslarzes, Charles André Vogel, Dieter Hahnloser, Martin Hubner, Nicolas Demartines and Fabian Grass in Health Services Insights
