# Supplementary material for: Cost Analysis of Outpatient Colectomy in a Tertiary Center: A Projected Medico-Economic Evaluation
Source: Health Serv Insights. 2024 Sep 24;17:11786329241284400. doi: 10.1177/11786329241284400 (PMC11439163; doi:10.1177/11786329241284400)
Supplement: sj-docx-1-his-10.1177_11786329241284400 – Supplemental material for Cost Analysis of Outpatient Colectomy in a Tertiary Center: A Projected Medico-Economic Evaluation [file sj-docx-1-his-10.1177_11786329241284400.docx]

**Online appendix.** DRG’s of the inpatient population eligible for an outpatient colectomy strategy

| DRG | Text | N (%) |
| --- | --- | --- |
| G18B | Small bowel and colon procedures or other stomach, esophagus and duodenum procedures without radiation therapy | 93 (69.4) |
| G18A | Small bowel and colon procedures or other stomach, esophagus and duodenum procedures without radiation therapy, with highly complex procedure, diagnosis or extremely severe complication management or CC | 12 (8.9) |
| G02B | Complex procedure or additional procedure on the stomach, esophagus and duodenum and congenital malformation, age < 2 years | 6 (4.5) |
| G02A | Intervention or diagnosis of small bowel and colon complications with extremely severe CC | 3 (2.2) |
| G13B | Other bowel procedures or enterostomy with extremely severe CC | 3 (2.2) |
| A90B | Complex treatment in U-IMC > 392/552 and < 1177/1105 disease specific points, or complex treatment requiring specific intensive care with complex treatment points in intermediate care unit | 2 (1.5) |
| G13Z | Other bowel procedures or enterostomy with extremely severe CC | 2 (1.5) |
| G17A | Other rectal resection without any particular intervention for digestive malignancy | 2 (1.5) |
| G38A | Complex intensive care treatment > 196/184 points or negative pressure therapy or acute geriatric rehabilitation beyond 21 days of treatment, with specific surgical intervention for any digestive organ disease or malfunction | 2 (1.5) |
| G70C | Other severe digestive organ condition or peritoneal catheter, age > 15 years old | 2 (1.5) |
| A90A | Complex treatment in U-IMC > 1176 / 1104 points or complex surgical procedure or > 8 radiation therapy sessions or early rehabilitation beyond 14 days of treatment for particular diseases | 1 (<1) |
| G09A | Interventions for hernias with extremely severe CC, more than one day of hospitalisation | 1 (<1) |
| G16Z | Complex rectal resection or deep pelvic wound dehiscence or interventions for malignancy or radiation therapy with significant abdominal intervention | 1 (<1) |
| G17Z | Other rectal resection without particular intervention | 1 (<1) |
| G38Z | Complication management procedures or complex intensive care treatment >196 / 360 points or specific negative pressure therapy, with particular surgical intervention for any digestive organ disease or malfunction | 1 (<1) |
| G86Z | Digestive organ disease or malfunction, one day hospital stay | 1 (<1) |
| N01A | Pelvic wound dehiscence in female patient, radical vulvectomy, particular hysterectomy or significant abdominal intervention with lymphadenectomy, or debulking for malignancy, with extremely severe CC or complex intensive care treatment > 119 points | 1 (<1) |
| Total |  | 134 |

DRG – Diagnostic Related Group; CC – Complication or Comorbidity; U-IMC – Unit of Intermediate Medical Care
